# Outbreak of carbapenemase-producing *Citrobacter farmeri* in an intensive care haematology department linked to a persistent wastewater reservoir in one hospital room, France, 2019 to 2022

**DOI:** 10.2807/1560-7917.ES.2024.29.14.2300386

**Published:** 2024-04-04

**Authors:** Marie Regad, Julie Lizon, Corentine Alauzet, Gabrielle Roth-Guepin, Caroline Bonmati, Simona Pagliuca, Alain Lozniewski, Arnaud Florentin

**Affiliations:** 1 Centre Hospitalier Régional Universitaire (CHRU)-Nancy, Département territorial d’hygiène et de prévention du risque infectieux, Vandœuvre-lès-Nancy, France; 2 CHRU-Nancy, Laboratoire de microbiologie, Vandœuvre-lès-Nancy, France; 3 CHRU-Nancy, Service d’hématologie, Vandœuvre-lès-Nancy, France; 4 CHRU-Nancy, Laboratoire de microbiologie environnementale, Vandœuvre-lès-Nancy, France; 5 Université de Lorraine, Département d'Hygiène, des Risques Environnementaux et Associés aux Soins (DHREAS), Faculté de Médecine, Vandœuvre-lès-Nancy, France; 6 Université de Lorraine, Institut national de la santé et de la recherche médicale (Inserm), Interdisciplinarité en Santé Publique Interventions et Instruments de mesure complexes (INSPIIRE), Nancy, France

**Keywords:** Drain outbreaks, environmental reservoir, carbapenemase-producing *Enterobacterales*, multidrug resistant organism, molecular typing, wastewater drains

## Abstract

In 2019–2022, a prolonged outbreak of oxacillinase (OXA)-48-producing *Citrobacter farmeri* due to a persistent environmental contamination, occurred in our haematology intensive care unit. In April 2019, we isolated OXA-48-producing *C. farmeri* from rectal samples of two patients in weekly screenings. The cases had stayed in the same hospital room but 4 months apart. We screened five patients who had stayed in this room between the two cases and identified a third case. Over the following 3 years, five other cases were detected, the last case in September 2022. In total, eight cases were detected: seven colonised with the bacterium and one infected with a lethal outcome. All cases stayed in the same hospital room. We detected OXA-48-producing *C. farmeri* from a shower, washbasin drains and wastewater drainage of the bathroom of the hospital room. Molecular typing confirmed that all *C. farmeri* isolates from the environment and the cases were indistinguishable. Despite bundle measures to control the outbreak, the bacterium persisted in the system, which resulted in transmission to new patients. A design defect in the placement of wastewater drains contributed to the persistence and proliferation of the bacterium. The room was closed after the last case and the bathroom rebuilt.

**Key public health message**

**What did you want to address in this study and why?**

Carbapenems are broad-spectrum antimicrobials, effective against many types of bacteria, including bacteria resistant to many other antimicrobials. We describe an investigation of an outbreak over a period of 3 years affecting eight patients and caused by a highly resistant carbapenemase-producing bacterium in a haematology department in a hospital in France.

**What have we learnt from this study?**

After extensive environmental investigations including wastewater and drains from all bathroom fixtures, we were able to identify a bathroom of a hospital room in the haematology department as the source of this outbreak. We have taken a combination of interventions including replacement of all bathroom drains and thus contained the outbreak.

**What are the implications of your findings for public health?**

Surveillance, including environmental sampling and typing of bacterial isolates, is important in the detection of outbreaks of carbapenemase-producing bacteria. Environmental monitoring must be part of the response to outbreak.

## Background

Carbapenemase-producing Enterobacterales (CPE) were detected in the 1990s and have spread worldwide during the 2000s [1]. In France, reports of hospital-acquired infections caused by emerging extensively drug-resistant bacteria are increasing sharply, rising from none in 2001 to 1,250 in 2016 [2]. In addition, the French incidence of CPE increased from 0.010 per 1,000 hospital days in 2019 to 0.023 in 2022 [3]. Currently, CPE are still considered emerging and there is a growing international concern about their spread. Infections caused by CPE are challenging in healthcare as they are restricting treatment options and increasing costs due to longer hospital stays [4,5].

In our hospital, infection prevention and control (IPC) measures, based on the recommendations of the French High Council of Public Health [6], are implemented and evaluated by the infection prevention and control team (IPCT). The measures include instructions for the management of patients who are suspected or confirmed carriers of multidrug-resistant organisms (MDRO). However, finding the source of the outbreak can be difficult and the compliance of hospital staff with IPC measures may sometimes be insufficient to contain an outbreak. Moreover, the healthcare environment, especially hospital wastewater drainage, has been a vehicle in transmission and washbasins d drains represent an invisible but proven potential source for MDRO [7-9].

## Outbreak detection

Between April 2019 and September 2022, we identified eight cases with oxacillinase (OXA)-48-producing *Citrobacter farmeri* in our hospital in France among patients treated in the haematology intensive care unit (HICU) and who stayed in the same hospital room. In April 2019, we isolated OXA-48-producing *C. farmeri* from rectal samples of two patients in weekly screenings. The cases had previously stayed in the same hospital room but 4 months apart. We screened five patients who had stayed in this room between the two cases and identified a third case. Over the following 3 years, five other cases were detected, the last case in September 2022.

The aim of this study was to describe the investigation and interventions to contain the prolonged outbreak of OXA-48-producing *C. farmeri.*

## Methods

### Setting

Our HICU is a 20-bed ward where patients with allogeneic haematopoietic stem cell transplantation and chimeric antigen receptor (CAR)-T-cell therapy are admitted. The HICU receives 20 patients per month with an average length of stay of 23 days. Each room has its own bathroom with a shower, toilet and washbasin. The rooms have a controlled environment: air quality (terminal high efficiency particulate air (HEPA) filters, 40 air exchanges per hour, overpressure) and water quality (0.2 µm terminal filters in all bathroom faucets and a washbasin with hygienic drain).

A plan for the monitoring of the hospital environment is drafted annually. According to the plan, the capacity and functionality of the air treatment is inspected and the surfaces of all hospital rooms in HICU and all intensive care unit (ICU) department are sampled. Monitoring is based on the French national monitoring standards, which specify the minimum requirements for air cleanliness of a clean room or controlled environment (European Union (EN) International Organization for Standardization (ISO) 14644). For HICU rooms, the requirements for limits of particles in the air and microbiological cleanliness are detailed in the ISO-5 Cleanroom standard. The microbiological criteria for a cleanroom are:  < 1 colony forming units (cfu)/m^3^ of total aerobic bacteria and the absence of filamentous fungi.

Bathroom showers are sampled once a year for *Legionella* and the sampling results assessed based on the French criteria of < 10 cfu/L for immunocompromised patients. Microbiological requirements for water from the washbasins are:  total count of aerobic bacteria < 1 cfu/100 mL (22°C for 72 h, ISO 8199:2018) and *Pseudomonas aeruginosa* < 1 cfu/100 mL (36°C for 48 h, ISO 16266:2006).

### Surveillance and management of multidrug-resistant organisms

According to the French national guidelines for prevention of transmission of vancomycin-resistant enterococci (VRE) and CPE, patients from a French overseas healthcare facility, patients with a history of hospitalisation abroad within the previous year, patients with past medical history of VRE or CPE carriage and patients with a history of contact with individuals with detection of VRE or CPE entering the ward must have a rectal swab on admission [6].

In our hospital, rectal swabs for MDRO (multidrug-resistant *Pseudomonas* species, imipenem resistant *Acinetobacter baumannii*, VRE and CPE) are taken from all patients admitted to the HICU within 48 h after admission and once weekly thereafter. In case of detection of VRE or CPE, these first-line control measures are undertaken (‘search and isolate’ strategy): active screening of all patients admitted to the unit and considered as contacts, implementation of contact precautions for colonised and/or infected patients and reinforcement of standard control measures according to the French guidelines [6,10].

The IPCT actively supports the unit with visits and phone calls to the ward and conducts additional investigations such as epidemiological, environmental or professional practice evaluations. The same healthcare team usually cares for patients in the unit with and without CPE. Rectal screening of CPE patients is repeated until three weekly negative results were obtained after hospital discharge or ward change. The IPCT also launches a retrospective search for contact patients and screens all patients treated in the unit 3 weeks before the admission of patients with CPE.

We consider an outbreak if at least two patients are colonised or infected with a MDRO with the same resistance pattern. In our hospital, we use the software programme Lumed-Zinc (https://lumed.ca/en/zinc/) for collection of microbiological and other patient data. Some alerts are planned for specific microorganisms, resistance profiles or cluster of the same microorganisms to allow an IPCT investigation as soon as a similar situation arises.

### Case definition

In this investigation, cases were defined as patients with OXA-48-producing *C. farmeri* detected between April 2019 and September 2022, ≥ 48 h after admission to Room X in HICU. We defined colonised cases as patients with OXA-48-producing *C. farmeri* detected in rectal swabs and infected cases as patients with OXA-48-producing *C. farmeri* detected from other specimens.

We collected data from the electronic medical records of the cases during the stay in Room X:

- Demographic variables, such as: age, sex and length of stay in Room X,
- Antimicrobial therapy (at least 2 days of treatment),
- Information on toilet independence,
- Microbiological samples, including the number(s) and date(s) of sampling.

### Microbiological investigations

Rectal swabs were sent to the clinical microbiology laboratory of our hospital. For detection of CPE, the samples were cultured on ChromID CARBA SMART plates (bioMérieux, Marcy-l’Étoile, France) and incubated at 35°C for 24–48 h. In case of bacterial growth, species identification was performed using the MALDI-TOF Vitek MS system (bioMérieux). Susceptibility testing for carbapenem resistance was performed by disk diffusion according to the European Committee on Antimicrobial Susceptibility Testing (EUCAST) guidelines [11]. For phenotypic detection of carbapenemase production, a multiplex lateral flow immunoassay (NG-test Carba 5; NG Biotech, Guipry, France) was carried out for the rapid identification of the five most prevalent carbapenemase families (New Delhi metallo-β-lactamases (NDM), *Klebsiella pneumoniae* carbapenemase (KPC), imipenemase metallo-β-lactamase (IMP), Verona integron‒encoded metallo-β-lactamase (VIM) and OXA-48-like). If this test was negative, a carbapenem inactivation method was used as a second method [12].

The hospital environmental laboratory analysed surface and water samples taken routinely in the HICU according to national recommendations [13]. Each environmental water sample was analysed for total count of aerobic bacteria, CPE, *Escherichia coli* and coliform bacteria (ISO 9308–1:2014).

For total count of aerobic bacteria from surfaces, the laboratory routinely used tryptone soya agar contact plates with disinfectant inhibitors (Polysorbate 80 and Lecithin) incubated at 30°C for 5 days. For detection of CPE, surface swabs were used and cultured on ChromID CARBA SMART plates. Plates were incubated at 35°C for 24–48 h and for an additional 72 h in case of no growth. Lactose Triphenyl Tetrazolium chloride (TTC) agar was used for the detection and enumeration of *E. coli* and coliform bacteria in water samples (250 mL/water point) using the membrane filtration method. Plates were incubated at 37°C for 48 h. Colonies of Enterobacterales obtained from water or surface samples were identified using the MALDI-TOF Vitek MS system (bioMérieux). In case of growth of *C. farmeri*, susceptibility testing to confirm carbapenem resistance and phenotypic detection of carbapenemases were performed as previously described for clinical isolates.

We used arbitrarily primed-PCR (AP-PCR) and Enterobacterales repetitive intergenic consensus-PCR (ERIC-PCR) for characterisation of the *C. farmeri* isolates. The extracted DNA (20 ng) was added to 50 μL of PCR mixture containing dNTPs, MgCl2 (50 mM), PCR buffer (10X), Taq polymerase (5 µ/μL) and 50 μM of ERIC-2 (5’-AAGTAAGTGACTGGGGTGAGCG) or AP primer 1 (5’-GGTTGGGTGAGAATTGCACG). The DNA was amplified by the MyiQTM Two‐Colour (Bio-Rad, Hercules, the United States (US)) device: 94°C for 5 min, followed by 40 cycles at 94°C for 1 min, 25°C for 1 min and 72°C for 2 min. The amplicons were then separated and run in gel electrophoresis (50 V, 1 h 35 min) [14,15]. The DNA fingerprints of the isolates obtained by both methods were visually compared by two observers and interpreted according to the following criteria: two isolates were assigned to distinct clonal groups if their patterns differed by more than one major band or two minor bands by at least one method [14,15].

## Results

### Epidemiological investigations

**Figure 1:**
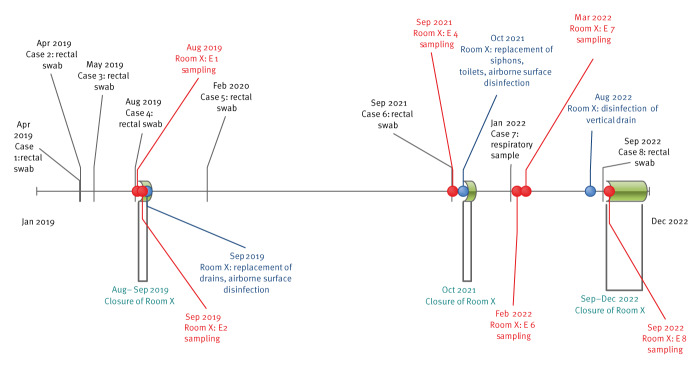
Timeline of detection of cases with oxacillinase-48 producing *Citrobacter farmeri* and control measures taken in an outbreak in an intensive care haematology unit, France, April 2019–September 2022 (n  =  8)

In April 2019, we detected the first two patients colonised with OXA-48-producing *C. farmeri* within the weekly routine rectal sampling in HICU (Figure 1). These patients (Case 1 and 2) stayed in the same room, Room X, 4 months apart, for 9 and 10 days, respectively, before detection of *C. farmeri*. We took rectal swabs from 52 contact patients of Case 1 and 2 but did not find any other cases. As contact patients, we considered patients who stayed in Room X between the stay of Case 1 and 2. We identified five other potential cases. Case 3 was detected in May 2019 and had stayed in Room X for 39 days between February and March 2019. The other four patients were screened three times a week but OXA-48-producing *C. farmeri* was not detected from the rectal swabs.

E: sampling of the hospital environment.

Detection of each case and sample type is coloured in black, sampling of the hospital environment (E) in red, control measures taken in blue and the closure of Room X in green.

In August 2019, Case 4 was detected during the second week of stay in Room X. Case 4 stayed in the room for 46 days. The sample taken 2 weeks before admission was negative (Table 1), OXA-48-producing *C. farmeri* was detected from the patient during the second week of stay. We did not detect secondary cases (screening three times a week) among patients identified as contacts (n = 27) to Case 4 or who stayed in Room X between Case 2 and 4 (seven patients with short hospital stay ranging from 1 to 11 days).

**Table 1:** Demographic and clinical characteristics of cases with oxacillinase-48-producing *Citrobacter farmeri* in an outbreak in an intensive care haematology unit, France, April 2019–September 2022 (n = 8)

Characteristics	Case identification number															
	1		2		3		4		5		6		7		8	
Length of stay (days) in Room X	9		42		39		54		36		39		9		31	
**Detection of *Citrobacter farmeri***																
Date	Apr 2019		Apr 2019		May 2019		Aug 2019		Feb 2020		Sep 2021		Feb 2022		Sep 2022	
Sample type	Rectal		Rectal		Rectal		Rectal		Rectal		Rectal		Tracheal aspirate		Rectal	
**Samples before detection of *Citrobacter farmeri***																
Number of sampling times	0						1		2		1		0			
Days between last negative sample and positive sample	Not applicable						5		7		7		Not applicable			
Time between first positive sample and stay in Room X (days)	108		10		87		46		17		12		15		21	
**Prior intravenous antimicrobial treatment (dose) and time (d) before detection of *Citrobacter farmeri***																
**Antimicrobial**	mg/d	d	mg/d	d	mg/d	d	mg/d	d	mg/d	d	mg/d	d	mg/d	d	mg/d	d
Amikacin	NT						2,000	3	1,400	4	NT		1,500	3	NT	
Amoxicillin	NT		1,500	7	NT		1,500	13	NT							
Cefepime	6,000	14	NT						6,000	14	6,000	3	6,000	3	6,000	2
Cefotaxime	NT												6,000	10	NT	
Ceftaroline	NT				1,800	7	NT									
Ceftazidime-avibactam	NT				6,000	5	NT									
Ceftriaxone	NT								2,000	10	2,000	21	NT		2000	7
Imipenem-cilastatin	NT		3,000	9	NT								3,000	7	NT	
Linezolid	1,200	14	1,200	14	1,200	21	1,200	14	1,200	8	NT		1,200	14	NT	
Meropenem	NT				3,000	14	NT									
Metronidazole	NT		1,500	14	NT				1,500	3	NT					
Piperacillin-tazobactam	NT		16,000	2	12,000	3	12,000	3	16,000	10	16,000	5	NT		16,000	14
Vancomycin	NT		2,000	10	2,000	4	NT		2,000	10	NT					
Time between first positive sample and first day of antimicrobial therapy in Room X (days)	108		47		42		37		12		9		15		14	

NT: not treated.

In February 2020, 6 months later, OXA-48-producing *C. farmeri* was isolated from rectal swabs of one patient (Case 5), and in September 2021 (19 months after the case 5), Case 6 was detected. These individuals stayed for 17 and 12 days in Room X, respectively, before detection of *C. farmeri*. Prior to detection, Case 5 had two negative rectal swabs and Case 6 had one. No secondary cases were detected among patients identified as contacts (n = 53 patients).

In February 2022, OXA-48-producing *C. farmeri* was detected from a tracheal aspirate of Case 7 after 19 months with no new cases. For the first time, the positive sample was not a rectal swab. Case 7 stayed in Room X for 9 days and had also a positive otorhinolaryngeal sample. This case had a lethal outcome but was empirically treated with imipenem-cilastatin for 9 days and died before treatment adjustment. No secondary cases were detected among the 26 contact patients of Case 7.

In September 2022, 7 months later, Case 8 was detected. Case 8 was hospitalised for 21 days in Room X before detection of OXA-48-producing *C. farmeri* from rectal swabs. No secondary cases were detected among the 21 contact patients of Case 8.

A description of the clinical characteristics of the eight cases is presented in Table 1. The average age was 58.4 years (standard deviation 20.1). Five of the cases were male and three were female. The patients stayed in Room X for an average of 37.5 days, and the time between the first positive sample and stay in Room X was in average 19 days.

All cases stayed in Room X and used the in-room toilet and shower. They all received antimicrobial therapy during their stay.

### Environmental investigations

Between April 2019 and September 2022, we took 10 surface samples from all HICU and ICU rooms as part of the routine monitoring and did not detect OXA-48-producing *C. farmeri* or other CPE.

In August 2019, after detection of Case 4, an environmental investigation (E 1) was conducted (Figure 1, Table 2). We took water and surface samples from Room X and analysed them without specific chromogenic medium. The sampling was done while Case 4 was staying in the room. All water samples were negative for OXA-48-producing *C. farmeri*. Among the surface samples taken (bathroom floor, rim of the bathroom washbasin, bathroom washbasin, chair, airlock washbasin, toilet, mattress edge, shower tray and telephone), only one, obtained from the toilet flange, was positive for OXA-48-producing *C. farmeri.*

**Table 2:** Environmental investigations and control measures taken in an outbreak of oxacillinase-48-producing *Citrobacter farmeri* in an intensive care haematology unit, France, August 2019–September 2022

Investigation number	Date	Patient in the room	Number of samples		Sample types		Selective medium^b^	Results
			Total	Positive	Surface samples	Water^a^		
E 1	Aug 2019	Case 4	9	1	Bathroom floor, rim of the bathroom washbasin, chair, airlock washbasin drain, toilet, mattress edge, bathroom washbasin drain, shower tray and telephone	Yes	No	Detection from toilet flange
E 2	Sep 2019 (12 days after previous investigations)	No	9	0	Mattress, telephone, toilet, anti-scar seat, airlock washbasin rim, shower wall/floor junction, rim of the bathroom washbasin, shower tray and toilet drains	Yes	No	Negative
E 3	Sep 2019 (8 days after previous investigations)	No	9	0		No	Yes	Negative
E 4	Sep 2021 (2 days after detection of Case 6)	Case 6	3	2	Shower and bathroom washbasin drains, toilet	No	Yes	Detection from bathroom washbasin drain and toilet flange
E 5	Oct 2021 (12 days after corrective measures)	No	3	0				Negative
E 6	Feb 2022 (16 days after detection of Case 7)	Yes (not identified as a case)	3	1		Yes	Yes	Detection from shower trap
E 7	Feb 2022 (23 days after detection of Case 7)	Yes (not identified as a case)	1	0	Ventilation ducts	No	Yes	Negative
E 8	Mar 2022 (36 days after detection of Case 7)	No	3	3	Wastewater vertical drain, toilet and shower drains	No	Yes	Detection from wastewater vertical drain, toilet and shower drains
E 9	Sep 2022 (15 days after detection of Case 8)	No	3	3	Toilet brush, bathroom washbasin drains and toilet	No	Yes	Detection from toilet brush, sink drain and toilet

E: environmental investigation.

^a^ Water samples: shower (before and after the filter), airlock and bathroom washbasin (before and after the filter).

^b^ Non-selective medium: tryptone soya agar; selective medium: ChromID CARBA SMART plates (bioMérieux, Marcy-l’Étoile, France).

We repeated the sampling in September 2019 (E 2) after discharge of Case 4 but all surface samples (mattress, telephone, toilet, anti-scar seat, airlock washbasin rim, shower wall/floor junction, rim of the bathroom washbasin, shower tray and toilet drains) and water samples were negative. In October 2019, a new sampling (E 3) was done. The bathroom surfaces were sampled as in E2. The samples were cultured on selective and non-selective media, but the target bacteria were not detected either from the surface or the water samples.

After the detection of Case 6 (September 2021), E 4 was performed while the room was occupied by Case 6. The sampling was done as in E 2, and OXA-48-producing *C. farmeri* was detected from two surface samples: toilet flange and washbasin drain. After Case 6 was discharged, we did a repeated sampling (E 5) but did not detect OXA-48-producing *C. farmeri*.

Following the detection of Case 7, we did a new sampling (E 6), analysed the samples using specific chromogenic media and detected OXA-48-producing *C. farmeri* from a sample from the shower drain. In addition, as the isolate from Case 7 was from an otorhinolaryngeal specimen, we collected surface samples from the ventilation ducts (E 7) of the hospital room but did not detect the bacterium. Finally, we took samples from the wastewater vertical drain of the shower, toilet and washbasin (E 8) and detected *C. farmeri* from the samples.

### Microbiological investigations

**Figure 2:**
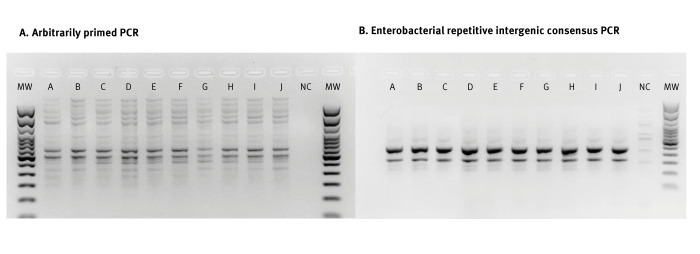
Characterisation of isolates of oxacillinase-48-producing *Citrobacter farmeri* from cases and hospital environment in an outbreak in an intensive care haematology unit, France, April 2019–September 2022

Isolates of OXA-48-producing *Citrobacter farmeri* from Case 1–7 (n = 7) and environmental isolates, one per sampling date, were characterised with AP-PCR and ERIC-PCR. The isolates were indistinguishable (Figure 2).

A: isolate from Case 1; B: isolate from Case 2; C: isolate from Case 3; D: isolate from Case 4; E: isolate from Case 5; F: isolate from Case 6; G: isolate from toilet flange sampled 16 Sep 2021; H: isolate from sink drain sampled 16 Sep 2021; I: isolate from shower trap sampled 15 Feb 2022; J: isolate from Case 7; MW: molecular weight; NC: negative control.

## Outbreak control measures

The outbreak investigation suggested an environmental origin, specifically the room environment, as a source. Consequently, the IPCT recommended airborne surface disinfection with acetic acid and hydrogen peroxide using Airinspace (Élancourt, France) equipment after discharge of Case 2 and change of the removal parts of the hygienic drains (Table 2).

In September 2019, after discharge of Case 4, we performed an airborne surface disinfection with acetic acid and hydrogen peroxide (using Airinspace), changed the washbasin and toilet drains and disinfected the washbasin drain with Oxy’floor (Anios, Lezennes, France). The room was closed until negative sampling results were obtained.

After Case 6 was discharged, the room was closed and thoroughly cleaned and disinfected with Airinspace. A new washbasin drain was installed, and the toilet was removed and replaced.

After detection of Case 7, we implemented a combination of chemical and mechanical measures. We used expanding foam (https://www.7darmor.fr/product/o-fresh-mousse.html) to clean and disinfect the bathroom. Then, we flushed the pipe with high pressure and heated water (70°C) and closed the room in October 2022 and reopened it in May 2023.

During the investigations, the hospital technical services informed us of two technical aspects of the room. Firstly, the two drains of the toilets and the bathroom washbasin were connected horizontally before vertically joining the wastewater pipe. Secondly, there was only one drainpipe for the toilet and the shower or washbasin, while in other rooms the pipes for the toilet and for the washbasin and shower were separate.

Despite the above-described corrective measures, Case 8 was detected, after which the room was closed. In January 2023, building of a new bathroom with an improved plumbing structure (fittings, bathroom washbasin, shower drain sealed on the slab) was started. As a preliminary step, the evacuation pipes were split into two with the creation of a new pipe for the toilets (between the fourth and fifth floors) and a new pipe for the shower/washbasin with a change of the washbasin drains. Samples taken after the installation of the new bathroom showed the persistence of MDRO in the shower drain, which had not been replaced in January. Consequently, the shower has been replaced.

## Discussion

The outbreak investigation suggested that transmission occurred via the persistence of OXA-48-producing *C. farmeri* in the washbasin drains and toilets. None of the cases was hospitalised at the same time. Therefore, we excluded direct transmission as a vehicle in this outbreak. Furthermore, patients in other rooms of the HICU were not colonised or infected with *C. farmeri*. Thus, transmission via a healthcare worker was not likely and the only common denominator was the stay in the same room (Room X). Moreover, characterisation of the isolates from cases and the environment showed that the isolates were indistinguishable.

Also, other researchers have shown transmission via persistent CPE in washbasin drains. Heireman et al. [16] and others detected MDRO infection or colonisation retrospectively with washbasin drains as a source [8,17-19]. Despite our interventions (decontamination and replacement of the washbasin drains), the bacterium could not be eradicated, and cases were detected during a period of more than 1.5 year. Detection of new cases several months apart despite corrective measures has been described before [9,20]. Carling et al. [9] reviewed 23 CPE outbreaks associated with hospital environment and concluded that these outbreaks lasted for an extended time, the majority (70%) for 2 years or more. Environmental reservoirs, in particular, contribute to occasional transmissions and persistent outbreaks. Eradicating CPE from washbasin drains tends to be problematic, and washbasins are optimal moist reservoirs for CPE to survive. Kotay et al. [21] analysed the resilience of biofilm in the horizontal drain of a laboratory wastewater system. More specifically, a biofilm promotes a persistent contamination of washbasin drain systems with carbapenem-resistant organisms via a multistage process. Parkes et al. [22] presented the effectiveness of various strategies to reduce the risk of washbasin-related infections. They explained that cleaning and disinfection alone are usually ineffective in eliminating microbial colonisation of washbasins due to biofilm. Nurjadi et al. also confirmed that a complete eradication is not possible with routine disinfection measures [23] but succeeded in preventing new cases of OXA-48 CPE after implementation of weekly autoclaved removable shower inserts. Vergara et al. [24] also reported that the replacement of contaminated washbasin drains alone did not stop occurrence of new cases.

We were able to detect a design flaw in the wastewater drains of Room X. The shower and washbasin drains were connected to each other via a horizontal drain, in contrast to other rooms in the haematology unit (with unconnected and vertical drainage), which could also explain biofilm formation and dispersion. A horizontal drainage system was removed in a Norwegian hospital outbreak [25]. We decided after the discovery of Case 8, when the drainpipe had been replaced, to completely replace all the plumbing elements in the room bathroom.

Inkster et al. [26] proposed in their commentary additional criteria that could be used assurances regarding safety for haemato-oncology units such as drain risk assessments validated cleaning protocols and a maintenance programme.

In our report, molecular typing was an additional aid in the outbreak investigation. Although PCR methods have a sufficient resolution power to discriminate isolates of Enterobacterales, the methods applied in our study are less informative than whole genome sequencing and is one of the limitations of our study. A second limitation, our previous monitoring software failed to alert us about the persistence of *C. farmeri* in the hospital room. This event led to an update of the software.

## Conclusion

The outbreak source may be undetected in routine sampling. It is essential to consider that preventive environmental measures are equally important in prevention as standard and contact measures. We need to focus on prevention more than ever with the proliferation of MDROs and the limited options for successful treatment.

We also recommend use of monitoring software to provide early warning and track such outbreak. Finally, this episode also demonstrated the importance of screening patients in haematology units on admission and on a weekly basis; to rule out or confirm nosocomial acquisition of microorganisms.
